# Efficiency of low dosage apatinib in post-first-line treatment of advanced lung adenocarcinoma

**DOI:** 10.18632/oncotarget.19908

**Published:** 2017-08-03

**Authors:** Da-Xiong Zeng, Chang-Guo Wang, Wei Lei, Jian-An Huang, Jun-Hong Jiang

**Affiliations:** ^1^ Department of Respiratory and Critical Care, The First Affiliated Hospital of Soochow University, Suzhou, China

**Keywords:** lung cancer, apatinib, adenocarcinoma, VEGFR-2, EGFR wild-type

## Abstract

Chemotherapy is the standard treatment of in advanced lung adenocarcinoma patients without driver mutation. However, few drugs could be selected when diseases progressed after second-line treatment. As a small molecule inhibitor of vascular endothelial growth factor receptor-2 (VEGFR-2), apatinib was suggested mainly using in advanced gastric cancer. In this study, we showed the results of apatinib as second-line to fourth-line treatment in EGFR wild-type advanced lung adenocarcinoma patients. 16 EGFR wild-type advanced lung adenocarcinoma patients were administrated apatinib (250-500 mg/d) orally. 3 patients showed partial response and 8 patients showed stable diseases response to apatinib, with a medium progression-free survival (PFS) of 4.4 month (2-10 months). The objective remission rate (ORR) was 18.75%(3/16). The total disease control rate (DCR) was 68.75% (11/16). The main toxicities were hypertension, hand-foot syndrome, proteinuria and thrombocytopenia which were tolerable and manageable. So, apatinib might be an optional choice for post-first-line treatment of EGFR wild-type advanced lung adenocarcinoma patients.

## INTRODUCTION

Lung cancer is the leading cause of cancer death worldwide. Non small-cell lung cancer (NSCLC) accounts for more than 70% of lung cancer. Epidermal growth factor receptor (EGFR) mutation and anaplastic lymphoma kinase (ALK) rearrangements were the most popular driver mutations in Asian NSCLC patients [1]. EGFR tyrosine kinase inhibitors (EGFR-TKIs) and ALK inhibitors have suggested as the first-line treatment for patients harboring EGFR mutations and ALK rearrangements respectively. However, there were about 45% NSCLC patients without genetic driver mutation [2]. In these advanced patients, platinum-based doublet chemotherapy is the first-line standard treatment option. Unfortunately, these patients would experience progression about 4-6 months after first-line chemotherapy [2]. The second-line treatment achieved a lower objective response rate (ORR) than first-line treatment. So, there were fewer drugs selected for post second-line treatment.

Angiogenesis is an essential step in tumor growth, development and metastasis [3]. Vascular endothelial growth factor (VEGF) signaling, activating VEGF receptor (VEGFR) and promoting angiogenesis, has been proved acting crucial role in tumor angiogenesis [4]. So, VEGF/VEGFR has become an important target in cancer therapy. In fact, agents targeting VEGF/VEGFR signaling have shown encouraging efficacy in several solid tumors. As the antibody agianst VEGF or VEGFR, bevacizumab or ramucirumab added to chemotherapy significantly improved progression-free survival (PFS) and overall survival (OS) in advanced nonsquamous NSCLC [5–6]. Bevacizumab has also been proved in ovarian cancer and colon cancer [7–8]. However, these agents were suggested adding to platinum-based doublet chemotherapy as first-line treatment for advanced nonsquamous NSCLC without driver mutation. Update, there was little evidence for anti-angiogenesis drugs monotherapy as post-first-line therapy in advanced lung adenocarcinoma.

As an oral small molecule inhibitor of vascular endothelial growth factor receptor-2 (VEGFR-2), apatinib has been demonstrated the efficiency and safety in breast and gastric cancer therapy[9–11]. Recent cases report studies indicated the efficiency of a higher dosage (500-825 mg/d) apatinib in 5 cases NSCLC patients [12–13]. In this pilot study, we showed the efficiency and safety of apatinib at a lower dosage (250-500 mg/d) in 16 advanced lung adenocarcinoma patients.

## RESULTS

The characteristics of all patients were shown in Table 1. Patients aged from 48 to 81 years (61.3±10.3 years). The male-to female ratio was 9:7. All Patients were diagnosed pathologically with advanced lung adenocarcinoma (IV stage) and EGFR wild-type. One female patient was harbored ALK positive. Most patients experienced first-line chemotherapy and second-line treatment before the oral apatinib therapy except for a female patient who was administrated apatinib for second-line treatment. In two male patients, apatinib was used as the fourth-line treatment. Three patients were administrated with apatinib 500 mg/d, others were administrated with 250 mg/d.

**Table 1:** Characteristics of 16 advanced lung adenocarcinoma patients before apatinib therapy

Patients	Age(ys)	Sex	Stage	EGFR	ALK	First-line treatment
1	51	F	IV(T_4_N_0_M_1b_)	-	-	Cisplatin+Pemetrexed
2	51	F	IV(T_4_N_0_M_1b_)	-	-	Carboplatin+Pemetrexed
3	61	M	IV(T_4_N_2_M_1a_)	-	-	Carboplatin+Pemetrexed
4	48	M	IV(T_3_N_2_M_1b_)	-	-	Carboplatin+Pemetrexed
5	54	M	IV(T_4_N_3_M_1b_)	-	-	Cisplatin+Pemetrexed
6	69	F	IV(T_4_N_0_M_1b_)	-	-	Cisplatin+Pemetrexed
7	67	F	IV(T_2_N_3_M_1b_)	-	-	Carboplatin+Pemetrexed
8	56	M	IV(T_2_N_2_M_1b_)	-	+	Carboplatin+Pemetrexed
9	81	M	IV(T_4_N_2_M_1b_)	-	-	Pemetrexed
10	73	M	IV(T_4_N_3_M_1C_)	-	-	Cisplatin+Pemetrexed
11	62	M	IV(T_3_N_2_M_1C_)	-	-	Carboplatin+Pemetrexed
12	64	F	IV(T_2_N_3_M_1b_)	-	-	Carboplatin+Pemetrexed
13	77	F	IV(T_4_N_0_M_1b_)	-	-	Pemetrexed
14	51	M	IV(T_4_N_2_M_1b_)	-	-	Cisplatin+Pemetrexed
15	62	M	IV(T_4_N_0_M_1b_)	-	-	Cisplatin+Pemetrexed
16	46	F	IV(T_4_N_3_M_1b_)			Cisplatin+Pemetrexed

**Figure 1:**
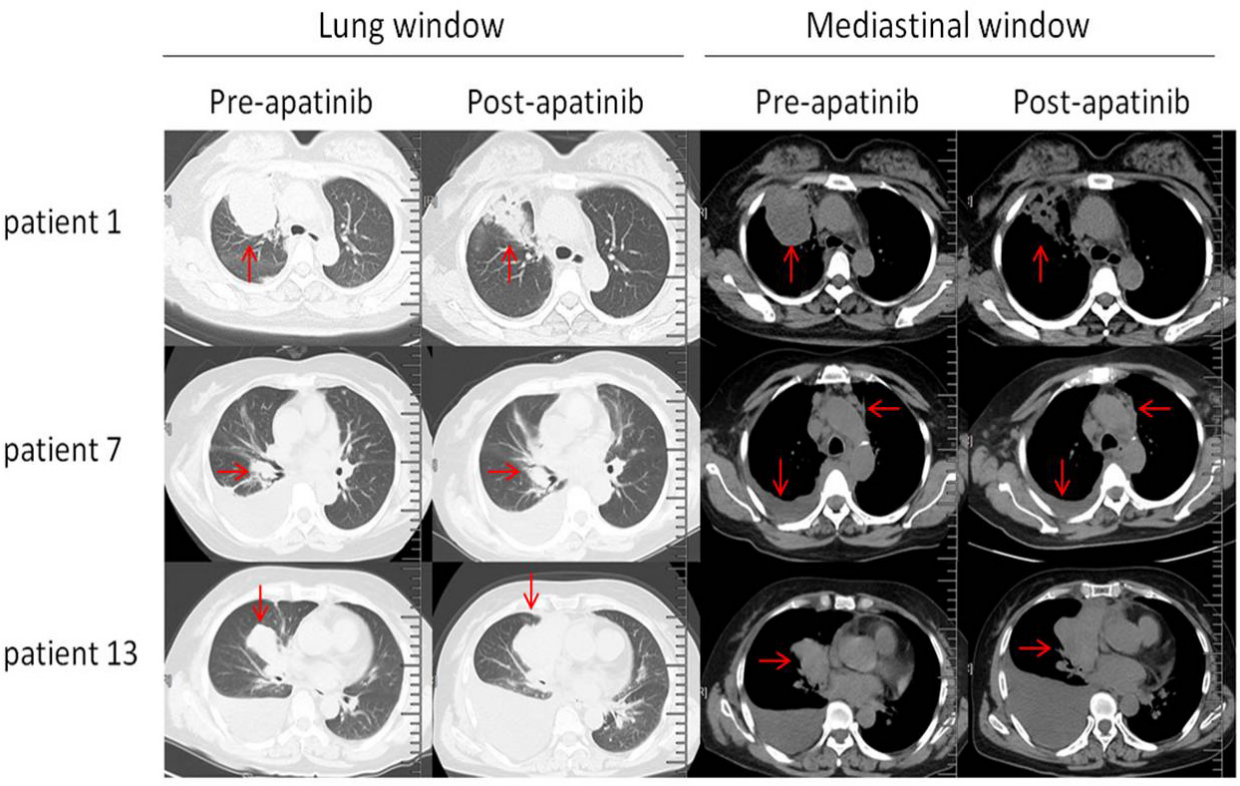
Representative CT images (lung windows and mediastinal windows) of three patients pre-apatinib and post-apatinib monotherapy CT images of patient 1 showed partial response of tumor in right upper lung (arrows). CT images of patient 7 showed stable disease response. Arrows showed the similarity of tumor in right middle lobe lung, enlarged lymph nodes and pleural effusion. CT images of patient 13 showed progressive disease response of tumor in right middle lobe lung and pleural effusion (arrows).

The results of tumor response were shown in Table 2. Figure 1 showed the representative CT images (lung windows and mediastinal windows) of some patients pre-apatinib and post-apatinib monotherapy. After apatinib monotherapy, 3 patients achieved partial response (PR), 8 patients achieved stable disease (SD), and 5 patient achieved progression disease (PD). The objective remission rate (ORR) was 18.75% (3/16). The medium PFS of apatinib monotherapy was 4.4±.7 months. The longest PFS was 10 months in a female patients (250 mg/d dosage), and the shortest PFS was only 1.5 months in a male patients (250 mg/d dosage). There were 4 patients achieved a PFS more than 5 months. The common adverse effects of apatinib treatment were hypertension (4/16), hand-foot reaction (2/16), proteinuria (2/16), hemoptysis (1/16), hoarseness (1/16), aleucocytosis (1/16) and thrombocytopenia (2/16). All adverse effects were Grade 1 to Grade 2, which were tolerable and manageable.

**Table 2:** The apatinib monotherapy and evaluation

Case	Apatinib and dosage	evaluation	PFS(months)	Advert effects
1	third-line, 500 mg/d	PR	7	hand-foot syndrome
2	second-line, 500 mg/d	SD	3.5	hypertension, aleucocytosis
3	third-line, 250 mg/d	SD	7	—
4	third-line, 250 mg/d	SD	2.5	—
5	third-line, 250 mg/d	SD	2	—
6	third-line, 250 mg/d	PR	10	hypertension
7	fourth-line, 250 mg/d	SD	3	hand-foot syndrome
8	third-line, 250 mg/d	SD	4.5	—
9	third-line, 250 mg/d	SD	1.5	hoarseness, hemoptysis
10	fourth-line, 250 mg/d	SD	5.5	hypertension
11	fourth-line, 250 mg/d	PD	-	—
12	third-line, 250 mg/d	PD	-	—
13	second-line, 500 mg/d	PD	-	thrombocytopenia, proteinuria
14	second-line, 250 mg/d	PD	-	—
15	second-line, 250 mg/d	PD	-	thrombocytopenia
16	third-line, 250 mg/d	PR	2	hypertension, proteinuria

## DISCUSSION

Platinum-based 2 drugs chemotherapy is the standard first-line treatment for advanced adneocarcinoma lung cancer without genetic driver. But the efficiency of chemotherapy has reached plateau period. There were few drugs selected for post second-line therapy. Recent studies showed that adding anti-angiogenic agents to chemotherapy significantly improved PFS and overall survival (OS) in advanced nonsquamous NSCLC [5–6]. As a monoclonal antibody against VEGF, bevacizumab showed benefit to OS in advanced nonsquamous NSCLC when adding to chemotherapy [5]. Another novel antibody against human VEGFR-2, ramucirumab had also been proved its combination with docetaxel significantly improved PFS and OS in second-line treatment of advanced NSCLC patients [6]. In this study, all patients received standard chemotherapy as first-line treatment. When diseased progressed, most patients received second-line chemotherapy. We did not selected bevacizumab or ramucirumab added to chemotherapy for first-line or second-line treament because of the patients’ poor economic status. However, there was few choices for these patients with EGFR wild type after second-line treatment.

Although drugs targeting VEGF/VEGFR signaling showing positive results in NSCLC treatment, the evidence was based on the combination of VEGF/VEGFR antibody and chemotherapy. Few investigations focused on the efficiency of anti-VEGF/VEGFR as post second-line treatment in cancer patients. As a small molecule oral drug, apatinib selectively inhibited VEGFR-2 activation. Recently, increasing evidence demonstrated the efficiency of apatinib in chemotherapy-refractory cancer patients [9–11]. In metastatic breast cancer patients, apatinib showed encouraging rates of disease stabilization and PFS [11]. In another Phase III study, apatinib signifcantly improved PFS and OS in chemotherapy-refractory advanced gastric cancer patients [9]. However, there remains no large scale clinical trial about apatinib in lung cancer treatment.

Recently, some cases reports focused on apatinib in lung cancer treatment. An *in vitro* study showed that apatinib inhibits cellular invasion and migration in lung cancer cells via suppressing RET/Src signaling pathway [14]. Two clinical cases reports studies demonstrated that apatinib prolonged PFS 2.8-6 months in advanced NSCLC without genetic driver mutation, even after second-line treatment [11–12]. Our study also indicated that advanced NSCLC patients could achieve a medium 4.4 months PFS by using oral apatinib alone as post second-line therapy. This result was in accordance to the previous case reports. By suppressing endothelial cells proliferation and migration, apatinib exerted anti-cancer effect in several solid tumors. Moreover, apatinib reverses cancer cells resistance by inhibiting the efflux function of multiple ATP-binding cassette transporters [15]. However, the precise mechanism in lung cancer remain need further study.

There are several points in our study different from previous reports. Firstly, we investigated more advanced adnocarcinoma lung cancer patients than previous cases reports. These would be more convincing for the usage of apatinib in NSCLC patients as post second-line treatment. Secondly, we used a lower dosage of apatinib than previous studies (250-500 mg/d *vs.* 500-850 mg/d). Interestingly, our patients not only achieved a similar PFS, but also suffered less adverse effects. Although the most suitable dosage of apatinib in advanced lung adenocarcinoma remain unclear, our results indicated that a lower dosage might be an optional choice.

In addition, our study investigated a lung adenocarcinoma patient with ALK-positive. Disease progressed after the first-line chemotherapy (carboplatin + pemetrexed for 4 cycles). Subsequent molecular detection showed ALK-positive. So crizotinib (250 mg bid) was used as second-line therapy. However, the patient showed crizotinib resistance after 5 months. As the second generation ALK inhibitor cannot get in the mainland of our country, the patients received docetaxel monotherapy as third-line treatment. Disease progressed 1 month later. So, apatinib was used as fourth-line treatment. This patient achieved a PFS of more than 3 months. So, this result indicated that apatinib might be a potential option for patient with crizotinib resistance when the second generation of ALK inhibitor cannot be got.

In the previous studies, high dose of apatinib (850 mg/d) was often accompanied with high rate of toxicity in gastric cancer patients, including hypertension (40.43%), proteinuria (27.66%), hand-footsyndrome (25.53%) andleucopenia (48.94%) [7]. In the 5 lung cancer patients reported in previous studies, apatinib (500-850 mg/d) also showed obvious side effects. In our study, we used a lower dose (250-500 mg/d) of apatinib which certainly resulting a lower rate of adverse effects. Furthermore, a lower dose of apatinib also received a total DCR of 68.75% (11/16) and medium PFS of 4.4 months as post second-line or third-line treatment. However, further investigation remains needed to confirm the most suitable dose of apatinib in advanced adenocarcinoma patients.

However, there are some limitations in our study. Firstly, the cases in our study were only 16 patients. A more large scale study with multiple centers would be more convincing. Secondly, the main endpoint of our study was PFS. Overall survival (OS) might be also another suitable endpoint. In addition, our study is retrospective study, not a random controlled trail. The design of a randomly and placebo-controlled clinical trail would confirmed the efficiency and safety of apatinib in advanced lung adenocarcinoma.

In conclusion, this pilot study indicated apatinib might be an option as post second-line treatment in advanced lung andenocarcinoma patients. However, several problems remain unclear, including the suitable dosage, the combination with chemotherapy, and the efficiency as first-line or second-line treatment.

## PATIENTS AND METHODS

This study was approved by the research ethics committee of the First Affiliated Hospital of Soochow University (ChiCTR-OPN-16009458). Informed consent was obtained from each patient. A total of 16 patients were enrolled in this study. All patients were pathologically diagnosed advanced lung adenocarcinoma (IV stage). The genetic driver mutation of EGFR and ALK were both detected. All patients experienced chemotherapy as the first-line treatment before the usage of apatinib. When diseases progressed after second-line or third-line treatment, patients were administrateda low dosage of oral apatinib (250-500 mg/d). 28 days (4 weeks) was defined as a therapy cycle.

The first chest CT scan was evaluated 4 weeks after apatinib treatment. Subsequently, tumor responses were evaluated every two cycle (8 weeks) according to the Response Evaluation Criteria in Solid Tumors (RECIST 1.1) using Chest imaging (CT scan). As described in RECIST 1.1, objective tumor responses included complete response (CR), partial response (PR), stable disease (SD), and progressive disease (PD). All the adverse effects were recorded, including proteinuria, hypertension, hand-foot reaction, hemoptysis, hoarseness.

## Abbreviations

(VEGFR-2): vascular endothelial growth factor receptor-2
(PFS): progression-free survival
(ORR): objective remission rate
(DCR): disease control rate
(NSCLC): non small-cell lung cancer
(EGFR): epidermal growth factor receptor
(ALK): anaplastic lymphoma kinase
(EGFR-TKIs): EGFR tyrosine kinase inhibitors
